# A Prospective, International, Multicentre Registry of Patients Undergoing Segmental Mandibular Defect Reconstruction After Mandibular Resection for Tumours and Drug-Induced Osteonecrosis: A Study Protocol

**DOI:** 10.3390/cmtr19010017

**Published:** 2026-03-23

**Authors:** Rüdiger M. Zimmerer, Tabea Pankow, Max Heiland, Julius Moratin, Wenko Smolka, Ali Modabber, Philippe Korn, Maria Mejia Nieto, Andreas Naros, Florian Thieringer, Rui Fernandes, Roderick Kim, Ashleigh Weyh, Eppo B. Wolvius, Mohemmed Khan, Andreas Thor, Marcel Ebeling, Takahiro Kanno, Alberto Pereira, Henrique Messias, Nils-Claudius Gellrich

**Affiliations:** 1 Department of Oral and Maxillofacial Surgery, University Hospital Tübingen, 72076 Tübingen, Germany; 2 Department of Oral and Maxillofacial Surgery, University Hospital Leipzig, 04103 Leipzig, Germany; tabea.pankow@medizin.uni-leipzig.de; 3 Department of Oral and Maxillofacial Surgery, Charité—Universitätsmedizin Berlin, Corporate Member of Freie Universität Berlin and Humboldt-Universität zu Berlin, Augustenburger Platz 1, 13353 Berlin, Germany; max.heiland@charite.de; 4 Universitätsklinikum Heidelberg, Mund-, Zahn-, Kieferklinik, 69120 Heidelberg, Germany; julius.moratin@med.uni-heidelberg.de; 5 Klinikum der LMU München, 80337 München, Germany; wenko.smolka@med.uni-muenchen.de; 6 Uniklinik RWTH Aachen, 52074 Aachen, Germany; amodabber@ukaachen.de; 7 Oral and Maxillofacial Surgery, Hannover Medical School, 30625 Hannover, Germany; korn.philippe@mh-hannover.de (P.K.); gellrich.nils-claudius@mh-hannover.de (N.-C.G.); 8 12 de Octubre University Hospital, 28041 Madrid, Spain; mmejianieto@hotmail.com; 9 Department of Oral and Maxillofacial Surgery, Tuebingen University Hospital, Osianderstrasse 2-8, 72076 Tuebingen, Germany; andreas.naros@med.uni-tuebingen.de; 10 Clinic for Oral- and Cranio-Maxillofacial Surgery, University Hospital Basel, 4031 Basel, Switzerland; florian.thieringer@usb.ch; 11 University of Florida College of Medicine, Jacksonville, FL 32209, USA; rui.fernandes@jax.ufl.edu; 12 John Peter Smith Health Network, Fort Worth, TX 76104, USA; roderickykim@gmail.com; 13 University of Illinois Hospital (UIHealth), Chicago, IL 60612, USA; aweyh@uic.edu; 14 Erasmus University Medical Center, 3075 EA Rotterdam, The Netherlands; e.wolvius@erasmusmc.nl; 15 Mount Sinai Hospital, New York, NY 10029, USA; mohemmed.khan@mountsinai.org; 16 Uppsala University, SE-75185 Uppsala, Sweden; andreas.thor@akademiska.se; 17 Department of Oral and Maxillofacial Surgery, University Hospital Ulm, 89081 Ulm, Germany; marcel.ebeling@uni-ulm.de; 18 Department of Oral and Plastic Maxillofacial Surgery, Military Hospital Ulm, 89081 Ulm, Germany; 19 Shimane University Hospital, Izumo 693-8501, Japan; tkanno@med.shimane-u.ac.jp; 20 Instituto Português de Oncologia de Lisboa, 1099-023 Lisbon, Portugal; albertorochapereira@sapo.pt (A.P.); jmessias@ipolisboa.min-saude.pt (H.M.)

**Keywords:** segmental mandibular defect, mandibular reconstruction, dental rehabilitation

## Abstract

Segmental mandibular resection may be indicated as a treatment in, for example, advanced stages of oral squamous cell carcinoma (OSCC). Osseous reconstruction of these defects is a fundamental part of static and dynamic masticatory rehabilitation, particularly when dental implants are required. The Segmental Mandibular Defect Reconstruction (SMDR) Registry aims to generate real-world evidence on SMDR through an international, prospective, multicentre case series designed as a registry. While OSCC is a common indication for segmental mandibular resection, the SMDR Registry also aims to capture outcomes for rarer mandibular conditions and the increasing number of collateral damage cases resulting from systemic medication therapies (antiresorptive drugs, immunotherapeutics) or irradiation, which may likewise lead to medication-related osteonecrosis of the mandible (MRONJ) or osteo(radio)necrosis with tumour-like segmental resection of the mandible, highlighting the value of an international database for these less frequent pathologies. Primary objectives are to describe the patient population and current treatment modalities, describe the outcomes and adverse events (AEs) for different treatment modalities, and identify potential predictors for successful autologous reconstruction of SMDs. Approximately 300 patients with a mandibular lesion resulting from bisphosphonate- and immunomodulatory drug-induced osteonecrosis of the mandible, ameloblastoma or osteosarcoma of the mandible, oral metastases related mandibular lesions indicated for segmental resection, or OSCC undergoing SMDR or intending to undergo one- or two-stage reconstruction will be prospectively recruited over a 36-month period. Baseline information, treatment details, and outcome measures will be documented. All treatments will be per the usual practice at participating sites. Outcome measures include clinical, patient-reported, and radiological outcomes; AEs related to the condition and/or treatment with a possible influence on the outcome will be recorded.

## 1. Introduction

Mandibular defects requiring reconstruction are common and occur after treatment of primary diseases, such as resection of benign or malignant lesions, inflammation, osteo(radio)necrosis, or as a result of trauma [1,2,3]. Management and reconstruction of segmental mandibular defects is challenging, as these defects can lead to severe dysfunction, facial disfigurement, and reduced quality of life, among others [4]. Segmental mandibular defect reconstruction (SMDR) is required to reconstruct the defect with the aim to restore function and aesthetics [5,6]. Manifold options exist for SMDR, including different graft types, donor sites, types of plates, and materials [7]. For transplanted bone grafts, the position, quality and quantity of transplanted bone into the mandible are crucial for later dental rehabilitation, which is one of the priorities in the restoration of functionality. After SMDR, it is known that the number of dentally rehabilitated patients is quite low (15–20%) [8,9,10,11], which is also reflected in our clinical experience, yet few studies describe the rates of and barriers to implant completion after resection. Pogrel et al. reported that 20% to 24% of patients who had osseous reconstruction of mandibular defects went on to have implants, but the reasons why patients did not have implants were not explored [12].

SMDR is further complicated by the debate around mandibular reconstruction timing, which is divided between proponents of one-stage surgery and two-stage surgery [4]. While immediate reconstruction performed in the same surgery as the tumour excision (one-stage surgery) allows for a shorter recovery period, it is technically demanding; in contrast, delayed reconstruction (two-stage surgery) allows for a period of observation to monitor recurrence and enables maturation of the wound bed before bone grafting [4,7]. However, it remains unclear which algorithm (one-stage or two-stage) has better outcomes in terms of position, quality and quantity of transplanted bone, and the effect of postoperative irradiation on transplanted bone. Continuous improvement in surgical resection and reconstruction techniques [13,14] has led to better patient outcomes. Modern treatment planning for patients undergoing segmental mandibular defect reconstruction after mandibular resection for tumours or medication-related osteonecrosis (MRONJ) integrates computer-aided design (CAD), advanced imaging software, segmentation techniques, customisable implants, and 3D models to enable precise, patient-specific surgical planning and reconstruction. Further, health-related quality of life (QoL) studies have shown significantly improved QoL in patients undergoing one-stage surgery [7]. Nevertheless, morbidity and incomplete rehabilitation still persist [15].

The disease variability and complexity of SMDR treatment options mean the effectiveness of the individual factors cannot be easily assessed. A registry gathering standardised information will provide medical and clinical evidence to assist clinical decision-making.

We have designed an international registry to prospectively collect data on patients undergoing reconstruction following segmental mandibular resection for OSCC, osteosarcoma, ameloblastoma, bisphosphonate- and immunomodulatory drug-induced osteonecrosis, and other metastatic mandibular lesions primary to OSCC. The goals are to describe the patient population, current treatment modalities, and outcomes and adverse events (AEs), as well as identify potential predictors for successful autologous reconstruction of segmental mandibular defects.

This registry aims to generate a database to drive future clinical research, generate focused study hypotheses, and answer unforeseen questions concerning the care and outcomes in this patient population.

## 2. Materials and Methods

### 2.1. Study Design and Setting

This is an observational study designed as an international, prospective, multicentre case series serving the function of a registry on patients undergoing the following segmental mandibular resection for OSCC, osteosarcoma and ameloblastoma, bisphosphonate- and immunomodulatory drug-induced osteonecrosis, and other metastatic mandibular lesions arising from malignancies originating in distant organs such as the lung, breast, kidney, and liver. Table 1 summarises the study sites currently included.

### 2.2. Objectives

Aside from collecting data into a large registry database on patients undergoing reconstruction following segmental mandibular resection for OSCC, osteosarcoma and ameloblastoma, bisphosphonate- and immunomodulatory drug-induced osteonecrosis, and other metastatic mandibular lesions rising from malignancies originating in distant organs such as the lung, breast, kidney, liver and prostate, the specific objectives are to (1) describe the patient population and current treatment modalities, (2) describe the outcomes and AEs for different treatment modalities, and (3) identify potential predictors for successful autologous reconstruction of segmental mandibular defects.

### 2.3. Study Procedures

Baseline information, treatment details, and outcomes will be collected in a customised, searchable database. All treatments will be performed according to the usual practice at participating sites; study-specific treatments, selection of devices or materials, and surgical or imaging techniques are not dictated in the study protocol, except for the prospective collection of a standardised set of data (demographic information including comedication, nicotine use, oral hygiene status, history of bisphosphonate treatment, immunomodulatory drug therapy, bone disease and comorbidities, tumour characteristics, adjuvant treatments, surgical parameters, osseous reconstruction details, additional surgeries, pathological assessment, clinical and patient-reported outcomes, and condition and treatment-related AEs). Post-treatment care and follow-up visits will be conducted according to the standard procedures at participating sites. Table 2 shows the inclusion and exclusion criteria.

### 2.4. Recruitment

A recruitment period of 3 years (36 months) is planned to enrol 300 eligible patients. Patient enrolment will be consecutive and competitive (i.e., with no limit in the number of patients enrolled at each site). Potentially eligible patients will be identified by an investigator and/or adequately trained member of the research team at the sites based on the patients meeting all the inclusion and none of the exclusion criteria, ideally after initial pathological/histologic diagnosis of mandibular involvement by oral tumours (such as OSCC, osteosarcoma, and ameloblastoma), bisphosphonate- or immunomodulatory drug-induced osteonecrosis, and mandibular lesions from metastatic diseases have been confirmed and the patients have been scheduled for surgery.

If a patient wishes to participate, a designated member of the research team will explain the informed consent process, including the purpose of the registry, the procedures, the potential risks and benefits, alternatives to participation, and data protection. Patients will also be informed about the possible future use of their de-identified data collected from the current registry for future research projects. Each patient choosing to participate will then sign and date an informed consent form. Consent will be obtained, where possible, before mandibular resection; if it is not possible (from the patient or the surrogate), the patient may provide consent after treatment up until the first standard-of-care follow-up visit, given the purely observational nature of the registry.

**Figure 1:**
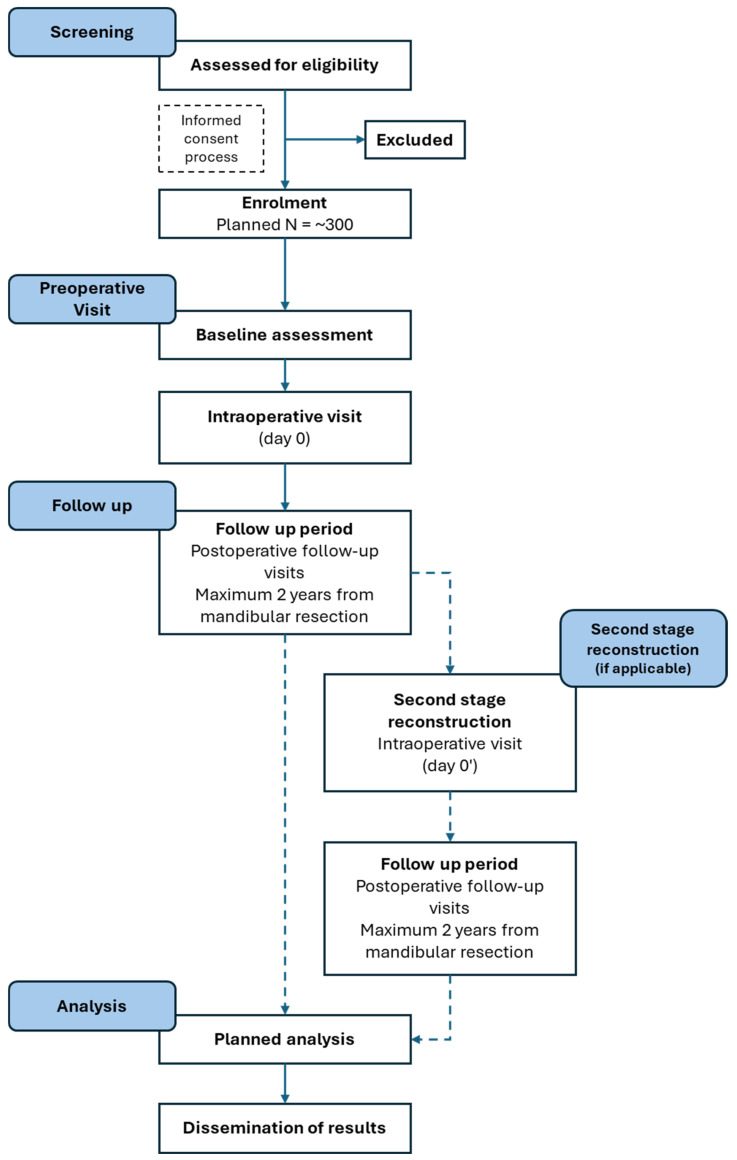
Planned patient flow.

All patients who provided consent will be allocated a unique patient number. Each site will keep an Identification List linking patient numbers with their personal information. This Identification List will always be kept safe and in a locked place. Sites will not be allowed to share the Identification List with any third party except for the sponsor representative, legal authorities, and EC/IRB, who may have access to the Identification List during monitoring or auditing activities performed on-site. Figure 1 shows the planned patient flow.

### 2.5. Data Collection

#### 2.5.1. Baseline Information

Demographic (sex and year of birth) and somatometric data (height and weight) will be recorded. Medical and lifestyle history data to be recorded will include data on OSCC, osteosarcoma of the mandible, ameloblastoma, oral metastases, and bisphosphonate- or immunomodulatory drug-induced osteonecrosis of the mandible. For OSCC, data on nicotine consumption and use of antiresorptive drugs (bisphosphonates or denosumab) will be collected. For osteosarcoma of the mandible, data on the history of genetic disorder, fibrous dysplasia and chronic osteomyelitis will be collected. For ameloblastoma, a history of dentigerous cyst, impacted teeth, injury to the mouth or jaw, pulpitis, or Gorlin–Goltz syndrome will be recorded. For oral metastases, a history of primary cancer in specified anatomic regions such as the lung, breast, kidney, or liver will be recorded. For osteonecrosis of the mandible, presence (yes/no) will be recorded, as well as the history of use of bisphosphonates and/or immunomodulatory drugs.

Comorbidities will be assessed using the Charlson Comorbidity Index [16,17]. Tumour characteristics to be recorded include the date of diagnosis, the tumour location, and the location of oral lesions due to metastases. Cancer staging will be documented according to the TNM system (8th edition [18]), including clinical and pathological staging. For this registry, for clinical staging of the data, the stage and cTNM method of assessment will be documented, and for pathological staging, the date and stage of pTNM will be documented.

For bisphosphonate- and/or immunomodulatory drug-induced mandibular osteonecrosis, the location as well as the staging (at risk or stage 0–3) will be documented, with “at risk” being the lowest category and “Stage 3” being the highest category. The classification based on Morton and Simpson’s theory will be documented as minor, moderate, or major [19].

If a panendoscopy is performed, the data and presence of simultaneous distant and/or local secondary carcinoma will be collected.

#### 2.5.2. Treatment Details

Both adjuvant treatment and surgical parameters will be collected. For adjuvant therapy, the dates (start and end) and types (radiotherapy, chemotherapy or targeted therapy and/or immunotherapy) will be recorded. Surgical parameters will be split into the tumour and segmental mandibular resection, the osseous reconstruction, and any additional surgeries related to mandibular reconstruction or dental implants. For tumour and segmental mandibular resection, the date and duration of surgery (skin-to-skin time in minutes) and length of hospital stay will be recorded. Additionally, for the resection details, the use of virtual surgical planning (VSP) (if used, including the use of 3D-printed biomodels and cutting guides) and the surgical approach will be documented. Further details of the mandibular defect (documented according to Brown’s classification [20]), as well as bone defect length, local soft tissue resection, and neck dissection, will be recorded.

For osseous reconstruction, the date of osseous reconstruction (if different from resection surgery) and reasons if not performed within 18 months after resection, as well as the use of VSP for osseous reconstruction (if used, including 3D-printed biomodels and cutting/drilling guides), will be recorded.

In cases of one-stage osseous reconstruction, the reasons for this choice will be documented, plus details of the bone and soft tissue flaps used, the fixation and osteosynthesis methods (including whether a 3D-printed biomodel from VSP was used), and supplementary bone graft, if applicable. Similarly, in cases of two-stage osseous reconstruction, the reasons for this choice will be documented, plus details of the first stage of mandibular resection and temporary alloplastic bridging with or without a solitary soft tissue flap. This will include details on the use of VSP for temporary alloplastic reconstruction, the use of independent soft tissue flaps, and fixation and osteosynthesis methods. Additionally, for the second stage of osseous reconstruction, the following aspects will be recorded: date and duration of surgery, length of hospital stay, use of VSP for osseous reconstruction, as well as details on use of bone and soft tissue flaps, fixation and osteosynthesis methods, and use of additional supplementary bone graft or collagenous membrane, if applicable.

Data on the additional surgeries related to mandibular reconstruction and dental implants to be recorded include date and duration of surgery, length of hospital stay, and indications for surgery.

### 2.6. Pathological Assessment

The pathological assessment will include staging according to pTNM, resection margins (if performed), histological type and subtype, grading (the poorest differentiation grading within the tumour will be documented), and human papillomavirus status (if tested).

### 2.7. Documented Visits

A summary of data to be collected at each visit is shown in Table 3.

Unscheduled visits may occur at any time due to, for example, a medical emergency; however, these visits will not be recorded in the registry, and information gathered between the scheduled registry visits will be documented at the next regular visit, except for the recording of any treatment-related AEs or if the patient drops out of the registry.

### 2.8. Termination of Participation

Participation in this registry may terminate early for reasons such as withdrawal of informed consent, the patient being found to be ineligible, investigator’s discretion (e.g., patient noncompliance), no osseous reconstruction with autologous bone performed within 18 months from resection, loss to follow-up, death, sponsor’s decision, or other reasons. Early terminations will be recorded in a dropout form, including the circumstances leading to the termination. All patient data collected before termination will be censored as of the day of the official withdrawal. Censored data will be included in the analysis, unless a patient explicitly requested removal of their data.

### 2.9. Outcome Measures

#### 2.9.1. Clinical Outcomes

Clinical outcomes (Table 2) to be assessed are:

- Neurological outcomes, including both sensory function in the mandible and the oral cavity, as well as motor function of cranial nerves that include the facial nerve (VII) marginal branch, hypoglossal nerve (XII), and accessory nerve (XI).
- Dental status, assessed according to the Eichner classification [21,22].
- Dental rehabilitation, including use, or potential use, of dental implants or equivalent within the reconstructed mandibular segment (date of placement, number and location of implants, type of healing for dental implants, time to function, dental implant status), as well as dentures (prosthodontic use) if the patient is edentulous or partially edentulous.
- Survival, including both recurrence-free survival (for OSCC, osteosarcoma, ameloblastoma and oral metastases but not for non-tumour conditions such as bisphosphonate- and/or immunomodulatory drug-induced osteonecrosis) and overall survival (applicable for OSCC, osteosarcoma, ameloblastoma, oral metastases, and non-tumour conditions such as bisphosphonate- and/or immunomodulatory drug-induced osteonecrosis).

#### 2.9.2. Patient-Reported Outcomes

Patient-reported outcomes (Table 2) to be assessed include the Oral Health Impact Profile (OHIP) [23], the EuroQoL five-dimension (EQ-5D-5L) [24] descriptive system, and patient satisfaction.

The OHIP was developed with the aim of providing a comprehensive measure of self-reported dysfunction, discomfort, and disability attributed to oral conditions [23]. The OHIP is concerned with impairment and three functional status dimensions (social, psychological, and physical). Respondents will be asked to indicate on a five-point Likert scale how frequently they experienced each problem within a reference period, for example, 12 months. Response categories for the five-point scale are “very often”, “fairly often”, “occasionally”, “hardly ever”, and “never” [25,26]. The OHIP-14 consists of 14 questions in which higher scores indicate worse outcomes. It has validated translations into several languages; those patients who speak a language for which no validated translation of the OHIP is available will not complete the questionnaire.

The EQ-5D-5L descriptive system comprises five dimensions: mobility, self-care, usual activities, pain/discomfort, and anxiety/depression. Each dimension has five levels: no problems, slight problems, moderate problems, severe problems, and extreme problems. The patient is asked to indicate their health state by ticking the box next to the most appropriate statement in each of the five dimensions. This decision converts into a one-digit number that expresses the level selected for that dimension. The digits for the five dimensions can be combined into a five-digit number that describes the patient’s health state [24].

The patient’s satisfaction with the overall treatment, facial aesthetics, and mouth dryness will be assessed using a five-point Likert scale. Both the EQ-5D-5L [24] and the patient satisfaction questionnaire are available in several languages.

#### 2.9.3. Radiological Outcomes

For the radiological outcomes, available images taken as part of the standard of care will be collected and evaluated centrally by the principal coordinating investigator. The following parameters will be assessed: mandibular dimensions, mandibular continuity, bone quality and quantity in the reconstructed segment, plate geometry/design, and precision of reconstruction. Images collected for this registry could be used for other analyses in the future.

### 2.10. Adverse Events

Because this is an observational registry, only AEs related to the condition and/or the treatments that might have an influence on the outcome will be collected. AE collection will commence as soon as the patient is enrolled. AEs do not need to be immediately reported unless they occur at a higher frequency and/or severity than that reported in the literature. The following AEs pertaining to the bone transplant (graft) and dental implants will be recorded: non-union or pseudarthrosis, malunion, bone sequestration, deep bone infection, osteo(radio)necrosis, total graft/transplant loss, insufficient anastomosis, and loss of dental implants within the reconstructed mandibular segment. The following AEs pertaining to implants and hardware will be recorded: hardware exposure, loosening and breakage, and screw loosening. For soft tissues, the following AEs will be recorded: wound dehiscence, superficial infection, loss of tissue flap or skin paddle, and neurosensory disturbances. General AEs to be collected include AEs related to adjuvant therapy; revision surgery for any other cause; any other AE related to the treatment of OSCC, osteosarcoma, ameloblastoma, osteonecrosis, and other oral metastases or mandibular reconstruction; and donor site morbidity for osseous flaps.

### 2.11. Statistical Considerations

The registry is exploratory in nature and thus not a hypothesis-driven clinical study. Therefore, no primary or secondary outcomes have been defined. Consequently, and in line with the exploratory nature of registries, no formal sample size or power calculation was performed. The proposed number of patients to be included in the registry (300) was estimated based on the number of patients who could feasibly be enrolled during the planned 3-year enrolment phase and was not based on a statistical hypothesis.

A detailed statistical analysis plan (SAP) will be prepared before the final analysis (i.e., the analysis from which the results will be summarised in the final registry report). The first final analysis will be done when 300 eligible patients have been enrolled and all follow-up visits have been completed. Generally, descriptive statistics will be generated for patient characteristics, clinical data, and outcomes recorded at standard of care scheduled follow-up assessments. Categorical outcomes will be summarised using frequency and percentage for each category. Continuous outcomes will be summarised using mean, standard deviation, median, interquartile range, and minimum and maximum. These summary statistics will also be presented according to clinically relevant categories, e.g., according to treatment pattern.

AEs will be summarised at both the patient level and the AE level. For the analysis on the patient level, multiple events of the same type will be combined for each patient, thus counting each patient with at least one AE only once. AE rates with 95% confidence intervals will be calculated. When calculating event rates, the denominator will be the total population size, irrespective of dropouts during follow-up.

Generally, all eligible patients who commenced treatment within the registry (i.e., having mandibular resection ≥2 cm and receiving reconstruction within 18 months after mandibular resection) will be included in the analysis. If a patient drops out, data collected up until that point will be included in the analysis. For specific research questions, only certain subgroups of patients may be relevant, and this will be predefined before the analysis. Depending on the amount and quality of the collected data, further appropriate statistics will be applied. Information on the analysis populations, the criteria for inclusion, the handling of missing data, and protocol violations will be defined and specified in the SAP.

### 2.12. Termination Criteria

The progress of the registry will be closely monitored, in particular the enrolment and safety aspects. Though there are no a priori termination criteria, the registry may be stopped for varying reasons (e.g., the registry fulfilling its purpose, poor quality of data, the registry being no longer relevant, and loss of funding/support to carry out the registry).

### 2.13. Current Status

The protocol in operation is version 3.0, dated 12 December 2024, as reflected in the manuscript, with protocol changes (inclusion and exclusion criteria) from the previous version. There are 13 participating sites from Europe, one from Japan, and four from North America, with ethics approval obtained from all sites. Sixteen sites have enrolled 138 patients, of whom 118 have thus far met the eligibility criteria. The first patient was enrolled in November 2022, and the data analysis is planned for May–September 2026.

### 2.14. Ethics and Dissemination

Data from each patient participating in the registry are documented in electronic case report forms (CRFs) and captured in the REDCap Cloud Electronic Data Capture system (http://www.redcapcloud.com) [27,28]. CRFs should be completed in a timely manner after the patient’s visit and are password-protected, with only authorised personnel having access. After termination of the registry, each site will receive an electronic copy of the data collected from the respective registry site. Because this study is observational, only standard-of-care procedures are performed, and only anticipated condition-related AEs are collected. Therefore, a data safety monitoring board has not been implemented. Data monitoring and cleaning will be performed on a regular basis to ensure data accuracy.

Ethics approval from the local ethics committee or institutional review board was obtained for each site before enrolling patients. The registry was designed and will be carried out according to current valid international guidelines (International Council for Harmonisation of Technical Requirements for Pharmaceuticals for Human Use (ICH) Guideline for Good Clinical Practice E6 [29] and ISO 14155 [30]), with the ethical position based on the Declaration of Helsinki, thus ensuring optimal protection of patient interests. The results of this study are intended to be published in peer-reviewed journals.

## 3. Discussion

This study protocol describes an international registry that will provide standardised information to support future clinical research and generate medical and clinical evidence on reconstruction following segmental mandibular resection for diverse pathologies, thereby assisting clinical decision-making. The study protocol was amended to widen the inclusion criteria to capture a larger number of patients with pathologies other than OSCC that necessitate reconstruction after segmental mandibular resection. This reflects the recent increase in defect reconstruction seen for non-primary cancer lesions in the mandible. Primary cancers such as lung, breast, prostate, or kidney cancers commonly metastasise to the mandible [31]. The positive outcome of increased survival of patients with such cancers due to advanced cancer treatments is countered by a potentially increased risk of developing metastases in the mandible. This, coupled with the complexity of different treatment options and constant technological innovation, has created a landscape in which it is difficult to obtain satisfactory evidence on the effectiveness of each treatment combination.

Many clinical questions remain in the present clinical landscape. These include the known limitations of donor sites; specifically, it is unclear how donor site morbidity influences the prolonged rehabilitation of patients undergoing reconstruction following segmental mandibular resection. Further, it is unclear whether the QoL of patients undergoing two-stage reconstruction is improved if a longer rehabilitation process is avoided. The primary endpoint of a reconstruction should be the ability to offer all patients a dental rehabilitation protocol, yet in the present treatment environment, only 15–20% of SMDR patients receive dental implants [8,9,10,11,12]. Moreover, mandibular reconstruction following tumour/mandibular resection poses notable challenges for treatment outcomes, particularly due to limitations inherent in both one-stage and two-stage osseous reconstruction techniques. One-stage reconstruction enables faster restoration but may result in suboptimal outcomes due to intraoperative difficulties in accurately contouring the graft, especially in complex defects, leading to impaired dental rehabilitation, aesthetic compromise, and increased flap failure risk. Two-stage reconstruction facilitates more effective treatment planning for complex defects and allows a period of bone adaptation before the final reconstruction but delays rehabilitation, increases infection and scarring risks, and often requires multiple interventions, adversely affecting recovery and overall clinical outcomes. Another aspect of this study will be to investigate the extent to which the principal goal of mandibular bone reconstruction can be realised and the cumulative surgical, prosthodontic, and logistical demands involved before achieving functional or even theoretically viable dental rehabilitation. This study has a two-year follow-up period; although it may not be sufficient to evaluate long-term outcomes, it will allow assessment of whether the reconstruction can be used for a dental implant and dental rehabilitation protocol. Any such dental rehabilitation should ideally take place within a time period of up to two years after the segmental defect reconstruction.

The amendment to the protocol, which involved broadening the inclusion criteria, has improved the recruitment rate. However, achieving the planned sample size still needs considerable time and represents a key limitation of the study. Additionally, the inclusion of a wider range of mandibular pathologies, such as osteosarcoma, ameloblastoma, oral metastases, and drug-induced osteonecrosis beyond the initially targeted OSCC, introduces heterogeneity that may complicate data analysis and interpretation, which can be considered a further limitation of the current protocol. On the other hand, the assumed large sample size enhances the statistical power of the study and increases the reliability and generalisability of the findings. Moreover, the inclusion of a diverse range of mandibular pathologies in addition to OSCC allows for a more comprehensive evaluation of the reconstructive protocol across different clinical scenarios. This heterogeneity may provide valuable insights into the protocol’s applicability and effectiveness in managing a broader spectrum of mandibular defects, thereby improving its clinical relevance and translational potential.

One strength of this registry study is that it will capture the resources invested to rehabilitate the addressed group of patients, with the registry-based approach allowing for the collection of real-world longitudinal data across diverse clinical settings, enhancing external validity and enabling the evaluation of outcomes in routine practice. By using best practice for each of the treatment arms instead of randomisation, the clinical pathway of each contributing medical centre is not affected; however, the non-randomised design means the study may be subject to unmeasured confounding factors, such as comorbidities and socioeconomic status. Moreover, bias may be introduced through differences in surgeon experience, case selection, and institutional resources, despite adherence to each centre’s standard of care. In the addressed patient population, implant-borne dental rehabilitation could or should have been achieved—but often has not been—to date. For these patients, those who received bony reconstruction after 18 months (potentially due to individual health issues, which restrict them from undergoing bony reconstruction) will not be included. The outcomes of this registry may provide important insights into dental rehabilitation in the different patient populations, and perhaps some of the reasons for these diverse barriers to dental rehabilitation may be examined. The multicentre aspect of the registry will provide a wide range of evidence across different healthcare landscapes.

The extensive scope of this multicentre registry will create a knowledge database enabling the development of research hypotheses and future research to provide evidence that may help address some of these open questions.

## Figures and Tables

**Table 1:** Current participating sites.

Name	Country
Uniklinik RWTH Aachen, Aachen	Germany
Charité—Universitätsmedizin Berlin, Corporate Member of Freie Universität Berlin and Humboldt-Universität zu Berlin, Department of Oral and Maxillofacial Surgery, Berlin	Germany
Hannover Medical School	Germany
University Hospital Heidelberg	Germany
University Hospital Leipzig	Germany
LMU University Hospital, Munich	Germany
University Hospital Tübingen	Germany
Military Hospital Ulm	Germany
Shimane University Hospital, Izumo	Japan
Erasmus University Medical Centre, Rotterdam	The Netherlands
Instituto Português de Oncologia de Lisboa, Lisbon	Portugal
12 de Octubre University Hospital, Madrid	Spain
Uppsala University Hospital, Uppsala	Sweden
University Hospital Basel, Basel	Switzerland
University of Illinois Hospital (UIHealth), Chicago	USA
Tarrant County Hospital, Fort Worth	USA
University of Florida College of Medicine, Jacksonville	USA
Mount Sinai Hospital, New York	USA

**Table 2:** Inclusion and exclusion criteria.

Inclusion Criteria	Preoperative Exclusion Criteria	Additional Exclusion Criteria
- Age 18 years and older - Patients undergoing reconstruction of mandibular defects of ≥2 cm in size following segmental resection of pathologic mandibles due to oral tumours (including OSCC, osteosarcoma, and ameloblastoma), medication-related osteonecrosis of the jaw associated with bisphosphonates or immunomodulatory agents, or metastatic disease from primary sites, such as the lung, breast, prostate, liver or kidney - Intention to undergo mandibular reconstruction with autologous bone using a primary (one-stage) or secondary (two-stage) approach - Informed consent obtained according to IRB/EC procedures	- Tumours affecting the condyle - Patients under palliative care - Previous extensive mandibular surgeries (including reconstructions, e.g., TMJ replacement) that may potentially confound the outcome measures	- No osseous reconstruction with autologous bone performed within 18 months of resection

EC, ethics committee; IRB, investigational review board; OSCC, oral squamous cell carcinoma; TMJ, temporomandibular joint.

**Table 3:** Data collection at each visit.

Assessment Parameters	Pre-, Intra-, and Postoperative Visits *									
	Visit 1	Visit 2	Visit 3	Visit 4 *	Visit 5 *	Visit 6 *	Visit 7 *	Visit 8 *	2nd Stage Recon	Post 2nd Stage Reconstruction Visits ^1^
	Screening/Preoperative	Intraoperative (Day 0)	Discharge	3 Months (±2 Weeks)	6 Months (±1) Month	12 Months (±2) Months	18 Months (±2) Months	2 Years (±3) Months	Intraoperative (Day 0′)	3 Months (±2 Weeks) 6 Months (±1) Month 12 Months (±2) Months 18 Months (±2) Months 2 Years (±3) Months
**Patient information/consent**	X									
**Eligibility**	X									
**Baseline characteristics**	X									
**Tumour/osteonecrosis of mandible characteristics**	X									
**Adjuvant treatment for the malignant conditions (OSCC, Osteosarcoma of mandible, Malignant ameloblastoma, Oral metastases) ^2^**			X							
**Surgical parameters**										
**Resection**		X								
**Osseous reconstruction ^3^**		X							X	
**Additional surgeries ^4^**				X	X	X	X	X		X
**Pathological assessment**			X							
**Clinical outcomes**										
**Neurological function**	X		X	X	X	X	X	X		X
**Dental status**	X		X		X	X	X	X		
**Dental rehabilitation**				X	X	X	X	X		X
**Survival**		X	X	X	X	X	X	X	X	X
**Patient-reported outcomes**										
**OHIP**	X		X	X	X	X	X	X		X
**EQ-5D-5L**	X		X	X	X	X	X	X		X
**Patient satisfaction**					X	X	X	X		X
**Adverse events**		X	X	X	X	X	X	X	X	X
**Radiological assessment ^5^**	X	X	X	X	X	X	X	X	X	X

* Postoperative FU visits with the defined time windows are calculated from the day of mandibular resection (i.e., day 0) and reconstruction (if the patient undergoes primary reconstruction).^1^ Post-second-stage reconstruction visits will be calculated from the day of secondary reconstruction (day 0). Patients will be followed up for a maximum of 2 years from the time of mandibular resection. ^2^ Information on adjuvant treatment for the malignant conditions may be available at a different timepoint and will be documented whenever it becomes available. ^3^ Osseous reconstruction can occur on the day of resection (one-stage reconstruction) or on a different day (two-stage reconstruction). ^4^ Additional surgeries may occur at any time after mandibular resection. ^5^ All images will be taken according to the local standard of care and will be collected. Assessments will be performed on available images.

## Data Availability

No data were generated or analyzed in this article, as this article describes a study protocol.

## Abbreviations

The following abbreviations are used in this manuscript:

AE	Adverse event
EC	Ethics Committee
EQ	EuroQoL
IRB	Investigational Review Board
OHIP	Oral Health Impact Profile
OSCC	Oral squamous cell carcinoma
QoL	Quality of life
SAP	Statistical analysis plan
SMDR	Segmental mandibular defect reconstruction
TMJ	Temporomandibular joint
TNM	Tumour, node, metastasis
VSP	Virtual surgical planning
